# Reconstituted Rice with Low-Glycemic-Index Potential as an Alternative to Polished Rice: Structural, Cooking, Nutritional, and In Vitro Digestibility Attributes

**DOI:** 10.3390/foods15183297

**Published:** 2026-09-17

**Authors:** Yongzheng Hu, Qiuxia Shen, Lin Yang, Mingyuan Li

**Affiliations:** 1 School of Food and Bioengineering, Xihua University, Chengdu 610039, China; 2 College of Light Industry and Engineering, Sichuan Technology and Business College, Dujiangyan 611830, China

**Keywords:** extrusion, functional reconstituted rice, low glycemic effect, nutritional characteristics

## Abstract

Polished rice is a global staple with a high glycemic index (GI), which makes it unsuitable for patients with diabetes. Functional rice reconstitution offers an effective technological approach for producing a low-GI nutritional staple. Konjac powder (KP), mulberry leaf extract (MLE), pueraria powder (PP), and resistant starch (RS) are functional ingredients that reduce the carbohydrate content of staple foods and attenuate postprandial blood glucose fluctuations. In this study, we developed three reconstituted rice formulations with low estimated glycemic index (eGI) using varying proportions of KP, MLE, PP, and RS, and investigated their nutritional composition, microstructure, cooking and textural properties, amino acid profile, volatile compounds, sensory quality, and in vitro starch digestibility. The functional ingredients increased dietary fiber and RS, formed a dense starch–colloid gel network with enhanced hydrogen bonding, improved cooking quality and texture, preserved the amino acid profile, enriched flavor, and achieved good sensory acceptability. Among the formulations, the MLE-high RS (MHR) formulation (80.0% rice, 9.0% KP, 0.5% MLE, 2.0% PP, and 8.5% RS) exhibited the lowest eGI as determined via in vitro digestion. Overall, this study provides theoretical and technical support for the development and industrial application of functional reconstituted rice with reduced starch digestibility.

## 1. Introduction

Rice is one of the most important food crops worldwide that provides dietary energy and protein, thereby occupying an irreplaceable position in human dietary structure [1]. However, the presence of rapidly digestible starch as the main component of polished rice, in which the dietary fiber-rich bran and germ are removed during processing [2], increases its glycemic index (GI). This leads to pronounced postprandial blood glucose fluctuations, which are unfavorable for individuals with diabetes [3]. Thus, the development of rice with low-GI potential holds broad market prospects.

Extrusion reconstitution technology allows for targeted formulation design, enabling the precise modulation of nutritional composition, processing characteristics, and the determination of the health functions of staple foods [4]. Consequently, the incorporation of functional ingredients to enhance reconstituted rice has emerged as a research hotspot. In particular, fortification with vitamins and minerals alleviates micronutrient deficiencies [5,6]. Moreover, the addition of dietary fiber from cereals, fruits, vegetables, and legumes into reconstituted rice through physical mixing or microencapsulation improves gut health, increases satiety, and regulates carbohydrate absorption [7,8]. In addition, fortification with phytosterols lowers cholesterol levels and atherosclerosis risk [9]. Reconstituted rice formed via extrusion processing exhibits a morphology similar to that of natural polished rice, allowing for the use of traditional cooking and achieving high consumer acceptability [10].

Safety, formability, and palatability are desirable characteristics for functional ingredients. Konjac powder (KP), resistant starch (RS), pueraria powder (PP), and mulberry leaf extract (MLE) are suitable ingredients that combine safety with physiological activity. They may be crucial in reducing the carbohydrate content of staple foods and in attenuating postprandial blood glucose fluctuations. The primary active component of KP is konjac glucomannan (KGM), which exhibits high viscosity, solubility, swellability, and good gel-forming properties in aqueous solutions [11]. It exhibits important health benefits in lowering cholesterol and triglycerides, and in improving blood glucose levels, intestinal motility, and immune function [12]. RS, a starch fraction that resists digestion and absorption in the small intestine of humans, is characterized by its low caloric content and high satiety [13]. Accordingly, RS reduces postprandial increases in blood glucose levels [14] and induces alterations in the gut microbiota, modifying the bile acid profile, reducing intestinal inflammation, and inhibiting lipid absorption [15]. PP is rich in pueraria polysaccharides and isoflavone bioactive compounds; it exhibits antioxidant, immunomodulatory, anti-inflammatory, and hypoglycemic properties [16]. MLE lowers blood glucose and cholesterol levels and enhances anti-inflammatory and antioxidant capacity [17,18]. Its active component (1-deoxynojirimycin) is a potent α-glucosidase inhibitor, which delays glucose absorption by inhibiting glucosidase and glycosyltransferase activities [19].

Although reconstituted rice for diabetes and obesity has been previously developed [20], systematic evaluation of its nutritional, structural, and sensory quality remains limited. Therefore, in this study, we developed three reconstituted rice formulations with different proportions of KP, MLE, PP, and RS, and investigated their basic nutritional composition, microstructure, cooking properties, nutritional quality, flavor profile, and sensory characteristics in comparison with those of natural rice (NR), which is the commonly consumed polished white rice. Furthermore, an in vitro digestion model was employed to elucidate the synergistic regulatory mechanism of these multicomponent formulations on starch digestion kinetics.

## 2. Materials and Methods

### 2.1. Materials

NR, PP, and white bread were purchased from local markets. KP was purchased from Chengdu Paite Biotechnology Co., Ltd. (Chengdu, China). MLE was purchased from Hunan Inner Pharmaceutical Ltd. (Changsha, China). RS (HI-MAIZE 260) was purchased from Foshan Rongen Food Co., Ltd. (Foshan, China). Glucose, α-amylase (from porcine pancreas), pepsin (from porcine gastric mucosa), pancreatin, and the glucose assay kit (R21648, GOPOD colorimetric method) were purchased from Shanghai Yuanye Bio-Technology Co., Ltd. (Shanghai, China). The RS assay kit was purchased from Megazyme International Ireland (K-RSTAR, Bray, Ireland). All chemical reagents used were of analytical grade.

### 2.2. Preparation of Reconstituted Rice

Three types of reconstituted rice were developed (Table 1). The proportions of raw materials in each formulation were determined via preliminary trials that aimed to balance material cost, extrusion behavior, product integrity, acceptable sensory quality, and adequate energy supply to meet the needs of daily human activities. Specifically, a series of gradient formulations was first screened based on the results of single-factor experiments. On the premise of keeping KP and PP relatively constant and minimizing the proportion of NR, RS was set as the primary variable added in a gradient manner, whereas MLE was introduced as a supplementary functional factor (either added or omitted) to construct three different formulations.

Raw rice was pulverized, sieved using an 80-mesh sieve (aperture 180 µm; particles passing through the sieve were collected), mixed with functional ingredients for 3 min, adjusted to 20% moisture, and further mixed for 8 min. The mixture was then extruded using a twin-screw extruder (DG75-II, Jinan DG Machinery Co., Ltd., Jinan, China) with zone temperatures of 90, 125, 145, 145, 125, and 90 °C (Zones I–VI), screw speed of 25 Hz, feeding speed of 15 Hz, screw length-to-diameter ratio of 28:1, and a die for rice production provided by the manufacturer. The extruded grains were dried in a hot-air oven (ZDKX, Jinan DG Machinery Co., Ltd., Jinan, China) at 60 °C to a moisture content <15%. The grains were then cooled to room temperature (20–25 °C) on a mesh sieve.

### 2.3. Determination of Basic Nutritional Components

The moisture, crude protein, crude fat, dietary fiber, and RS content was determined via drying (GB 5009.3-2016 [21], National Food Safety Standard—Determination of Moisture in Foods), Kjeldahl (GB 5009.5-2016 [22], National Food Safety Standard—Determination of Protein in Foods), Soxhlet extraction (GB 5009.6-2016 [23], National Food Safety Standard—Determination of Fat in Foods), enzymatic-gravimetric (GB 5009.88-2014 [24], National Food Safety Standard—Determination of Dietary Fiber in Foods), and an RS assay kit (K-RSTAR, Megazyme International Ireland, Bray, Ireland), respectively. All the above national standards were valid when the experiments were performed. GB 5009.5-2016 [22], GB 5009.6-2016 [23] and GB 5009.88-2014 [24] have since been superseded by GB 5009.5-2025, GB 5009.6-2025 and GB 5009.88-2023, respectively.

### 2.4. Scanning Electron Microscopy

Intact rice grains with plump morphology and regular, complete shapes were selected, cut into approximately 1 mm thick sections using a sharp stainless steel blade, and gently adhered to conductive tape. The samples were sputter-coated with a gold layer for 90 s using an ion sputtering apparatus and subsequently observed via scanning electron microscopy (SEM; Inspect, FEI Company, Hillsboro, OR, USA) at magnifications of ×200 and ×3000.

### 2.5. Fourier Transform Infrared Spectrometry

A tablet prepared from 2 mg of dried, pulverized sample and 200 mg of KBr was analyzed via Fourier transform infrared (FT-IR) spectroscopy (Nicolet 380, Thermo Fisher Scientific, Waltham, MA, USA) over a range of 400–4000 cm^−1^ at a resolution of 4 cm^−1^ with 64 scans. A blank KBr tablet was used as a reference, and air was used as background.

### 2.6. Determination of Cooking Properties

Water absorption (WA), volume expansibility (VE), and rice gruel iodine blue values for rice were determined according to the procedures described by Liao et al. [25] and Yang et al. [26] with slight modifications. Briefly, 7.0 g (m_1_) of rice was heated in 70 mL of boiling water for 20 min, drained, and weighed (m_2_). The volumes of samples before (V_1_) and after (V_2_) cooking were determined based on water displacement. WA and VE were calculated using Equations (1) and (2), respectively.

Having cooled the collected rice gruel to room temperature (20–25 °C), its pH was measured using a pH meter. The gruel was then diluted with 100 mL of water, and a 15-mL aliquot of this suspension was transferred to a centrifuge tube and centrifuged at 1710 × *g* for 10 min. A sample of the resulting supernatant (1 mL) was added to 50 mL of distilled water, followed by 5 mL of 0.5 mol/L HCl and 1 mL of iodine reagent (2 g/L), and then diluted to 100 mL. Absorbance was measured at 620 nm for determination of the iodine blue value.

(1) $$WA(\%)=\frac{m_{2}}{m_{1}}\times 100\%$$

(2) $$VE(\%)=\frac{V_{2}}{V_{1}}\times 100\%$$

### 2.7. Texture Profile Analysis

For texture profile analysis (TPA), an appropriate amount of rice and tap water were added to a rice cooker (SF40FC875, Zhejiang Supor Household Electrical Appliance Manufacturing Co., Ltd., Hangzhou, China) at a ratio of 1:1.1 (m/V), cooked on standard mode for 12 min, and then kept warm for 5 min. Three morphologically similar rice samples were arranged radially on the platform of a texture analyzer (TA-XT PLUS, Stable Micro Systems Ltd., Surrey, UK) [27]. The following parameters were used: probe, P35; pre-test speed 1.0 mm/s; test speed 0.5 mm/s; post-test speed 1.0 mm/s; compression ratio 70%; and trigger force 5 g.

### 2.8. Amino Acid Content

The samples were hydrolyzed according to GB 5009.124-2016 (National Food Safety Standard—Determination of Amino Acids in Foods) [28], and the amino acid composition was determined using a fully automatic amino acid analyzer (L-8900, Hitachi, Ltd., Tokyo, Japan).

### 2.9. Volatile Compound Analysis

Volatile compounds were analyzed using headspace solid-phase microextraction coupled with gas chromatography-mass spectrometry (GC-MS, 2020NX, Shimadzu Corporation, Kyoto, Japan) following the method of Kang et al. [29] with modifications. A 10-g sample was placed in a headspace vial and mixed with distilled water (1:1.1, *w*/*v*). Having sealed the vial, it was heated in a water bath and equilibrated at 70 °C. A 75-µm carboxen/polydimethylsiloxane fiber (Supelco, Bellefonte, PA, USA) on a holder (57330-U) was exposed to the headspace for 30 min, after which, the adsorbed volatiles were desorbed at 250 °C for 5 min in the GC inlet. Separation was performed on a DB-5MS column (30 m × 0.25 mm, 0.25 µm) with helium carrier gas at 1.0 mL·min^−1^. The oven program was set as follows: 40 °C (2 min), increased to 90 °C at a rate of 6 °C·min^−1^, increased to 250 °C at a rate of 10 °C·min^−1^, and held for 10 min. The mass spectrometer was operated in electron ionization mode (70 eV), with a full scan (33–450 amu), an ion source at 250 °C, and an interface at 280 °C. Compounds were identified by matching with the NIST17 library (similarity index > 80) and retention times; relative content was determined via peak area normalization.

### 2.10. Determination of Sensory Evaluation

The sensory evaluation was conducted according to GB/T 15682-2008 [30] (Inspection of grain and oils—Method for sensory evaluation of paddy or rice cooking and eating quality, valid at the time of experiments and later superseded by GB/T 15682-2025) with slight modifications. Rice samples were cooked with deionized water at a 1:1.1 (*w*/*v*) rice-to-water ratio in an electric rice cooker (SF40FC875, Zhejiang Supor Household Electrical Appliance Manufacturing Co., Ltd., Hangzhou, China) and then kept warm for 5 min. Cooked samples (20 g per portion) were labeled with three-digit random codes and presented in a fully randomized order.

The panel consisted of 15 food science majors (aged 20–26 years) recruited with no taste/olfactory disorders or rice-related food allergies. All panelists received training on evaluation dimensions and scoring rules before testing and scored samples in accordance with the criteria listed in Table 2. Purified water mouth rinsing and a 1 min rest interval were required between samples. All tests were performed in individual, odor-free sensory booths under uniform white light and quiet ambient conditions.

### 2.11. In Vitro Digestion and Calculation of Estimated GI

The digestive juices were prepared, and in vitro digestion was conducted in accordance with the procedure described by Minekus et al. [31]. The rate of starch hydrolysis (HR) was calculated using Equation (3) as follows:

(3) $$HR/\%=\frac{m_{g,t}}{m}\times 0.9\times 100,$$

where $m_{g,t}$ is the weight (mg) of glucose in the hydrolysate at time t, m is the weight (mg) of total starch in the sample, and 0.9 is a factor for the conversion between glucose and starch.

The hydrolysis curve was obtained by plotting HR against time, and first-order kinetics [Equation (4)] were used for curve fitting [32] as follows:

(4) $$C_{t}=C_{\infty }\left(1-e^{-kt}\right),$$

where $C_{t}$ and $C_{\infty }$ are the rates of starch hydrolysis at time t and the hydrolysis endpoint (%), respectively; k is the kinetic constant; and t is the hydrolysis time (min). The area under the curve from 0 to 180 min was calculated via integration. Using white bread as a reference, the hydrolysis index (HI) was calculated using Equation (5) as follows:

(5) $$HI=\frac{AUC_{sample}}{AUC_{white\;bread}}\times 100$$

Values for the estimated glycemic index (eGI) were determined using Equation (6) [33] as follows:

(6) eGI=0.549HI+39.71

### 2.12. Data Analysis

The results were processed using Microsoft Excel 2024 and are presented as mean ± standard deviation for at least triplicate determinations, with all replicates independently sampled from different time points within the same extrusion batch. The OriginPro software (Version 2021) was used to determine significant differences, perform fitting and integration, and to generate figures. Multiple comparisons were performed using Tukey’s method, for which a confidence level of p<0.05 was considered statistically significant.

## 3. Results and Discussion

### 3.1. Basic Nutritional Components

**Figure 1:**
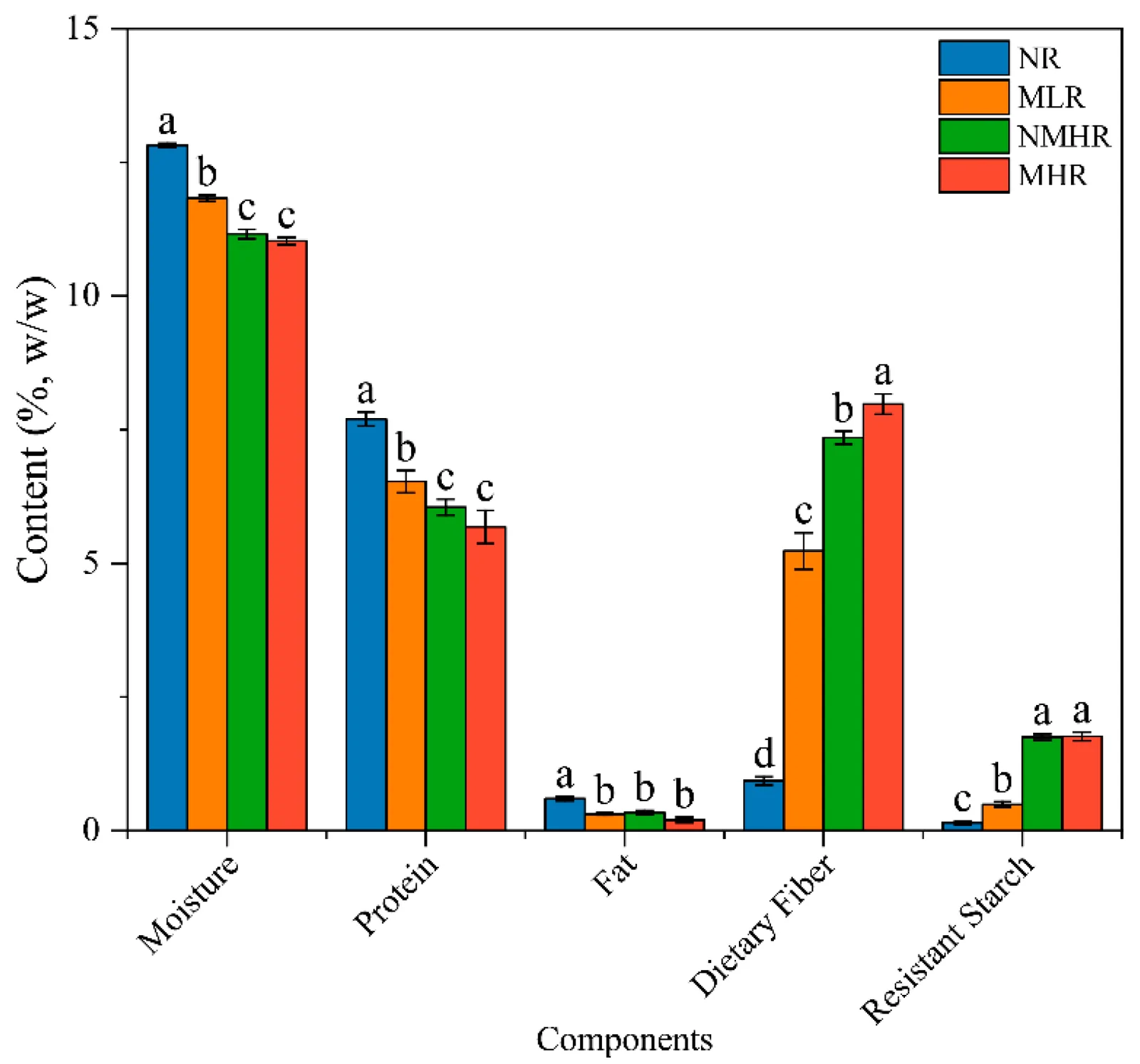
Basic nutritional components of NR and reconstituted rice. NR, natural rice; MLR, mulberry leaf extract–low-resistant starch rice; NMHR, non-mulberry leaf extract–high-resistant starch rice; MHR, mulberry leaf extract–high-resistant starch rice. Different letters indicate significant differences (*p* < 0.05).

The basic nutritional composition of NR and reconstituted rice is shown in Figure 1. The protein and fat content of NR was substantially higher than that of the three reconstituted rice samples. This was primarily because the background levels of crude protein and crude fat in the functional ingredients were much lower than those in rice; hence, this resulted in a dilution effect. The high temperature and pressure during extrusion promote the formation of complexes between proteins, fats, and starch [34,35], which may also induce oxidative degradation of fats and protein denaturation and aggregation, further reducing the protein and fat content. This mechanistic interpretation is supported by Gao et al. [3], who further reported that high pressure at the die could induce fat separation from the material matrix, representing an additional contributor to the reduced fat content. The dietary fiber and RS content increased substantially with the addition of functional ingredients. The dietary fiber content of NR was only 0.93%, whereas that of MHR reached the highest value of 7.98%, which is 8.58-times higher than that of NR. No pronounced difference in the RS content was observed between NMHR and MHR; however, both were markedly higher than those in MLR and NR (>10-times that in NR). The increases in dietary fiber and RS are mainly attributable to the insoluble dietary fiber and plant polysaccharides in KGM and PP, as well as to the addition of exogenous RS.

### 3.2. SEM Analysis

**Figure 2:**
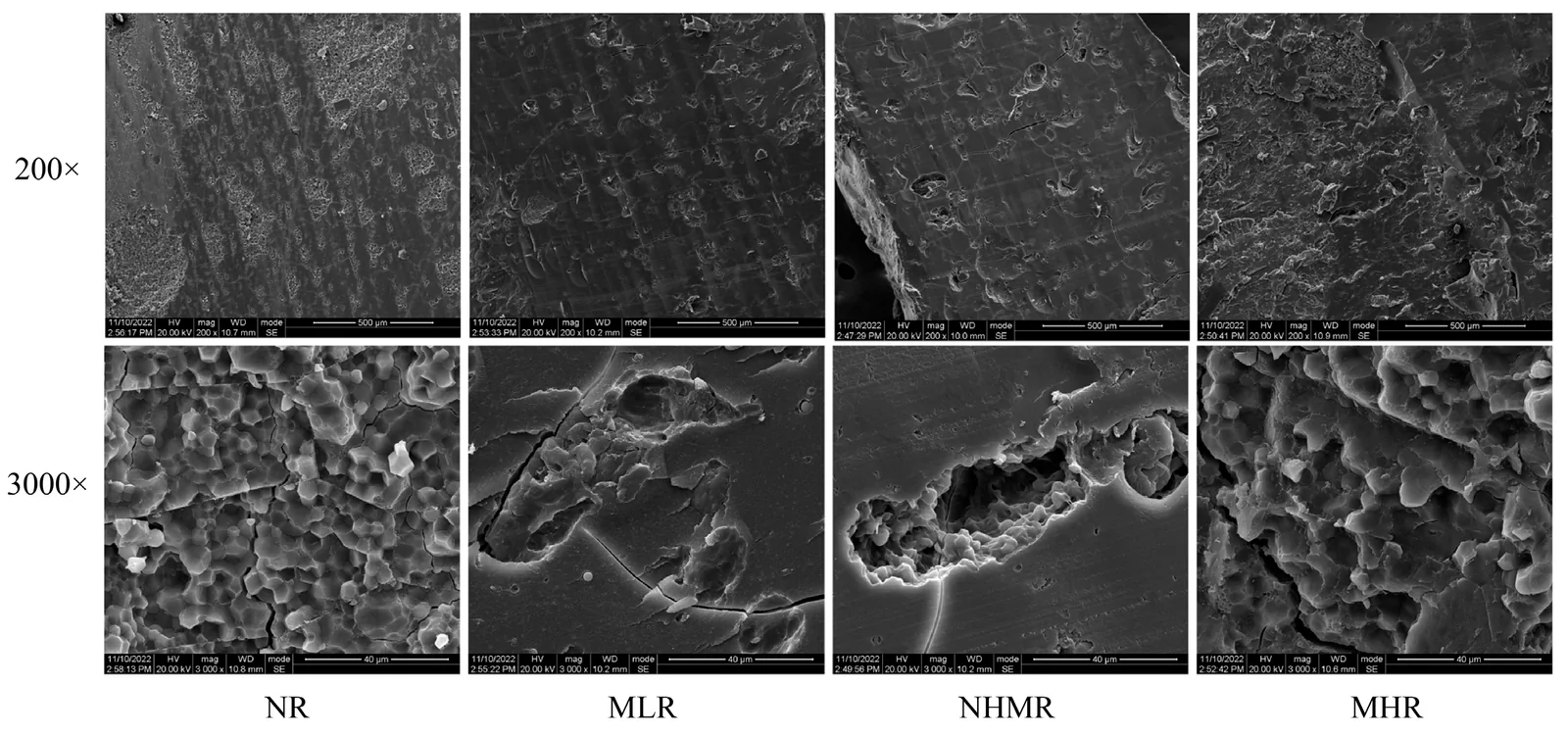
Microstructure of NR and reconstituted rice. NR, natural rice; MLR, mulberry leaf extract–low-resistant starch rice; NMHR, non-mulberry leaf extract–high-resistant starch rice; MHR, mulberry leaf extract–high-resistant starch rice.

The microstructure of the rice samples is presented in Figure 2. At low magnification, NR showed a smooth, intact surface, whereas MLR, NMHR, and MHR maintained grain morphology similar to that of NR. This indicated good formability; however, surface roughness increased slightly in MLR, and pitting and roughness increased with the RS content in NMHR and MHR. At high magnification, NR displayed densely packed starch granules with distinct edges, whereas the reconstituted rice groups lacked such granular characteristics. MLR and NMHR exhibited pores or fissures on smooth planar surfaces, whereas MHR showed cracks and no discernible granule outlines. These changes are attributable to the high temperature, high pressure, and shear forces generated during extrusion.

High temperature and pressure transform solid materials into a molten state. This promotes uniform fusion among components and results in regionally smooth planar surfaces after extrusion [3]. Furthermore, high temperature and shear force caused starch swelling and disruption, which resulted in rougher reconstituted rice particles and significant differences in starch microstructure compared with that in NR [36]. Water vapor that is not promptly expelled after high-temperature induction leads to the formation of internal voids upon cooling and setting. Additionally, the fiber components in the functional ingredients disrupted matrix continuity and stability, leading to drastic escape of water and air, ultimately rendering the product porous, rough, and non-uniform [37,38]. No obvious phase separation or particle agglomeration was observed in the reconstituted rice. This indicates good compatibility between the functional ingredients and the starch matrix, as well as uniform dispersion.

### 3.3. FT-IR Spectroscopy

**Figure 3:**
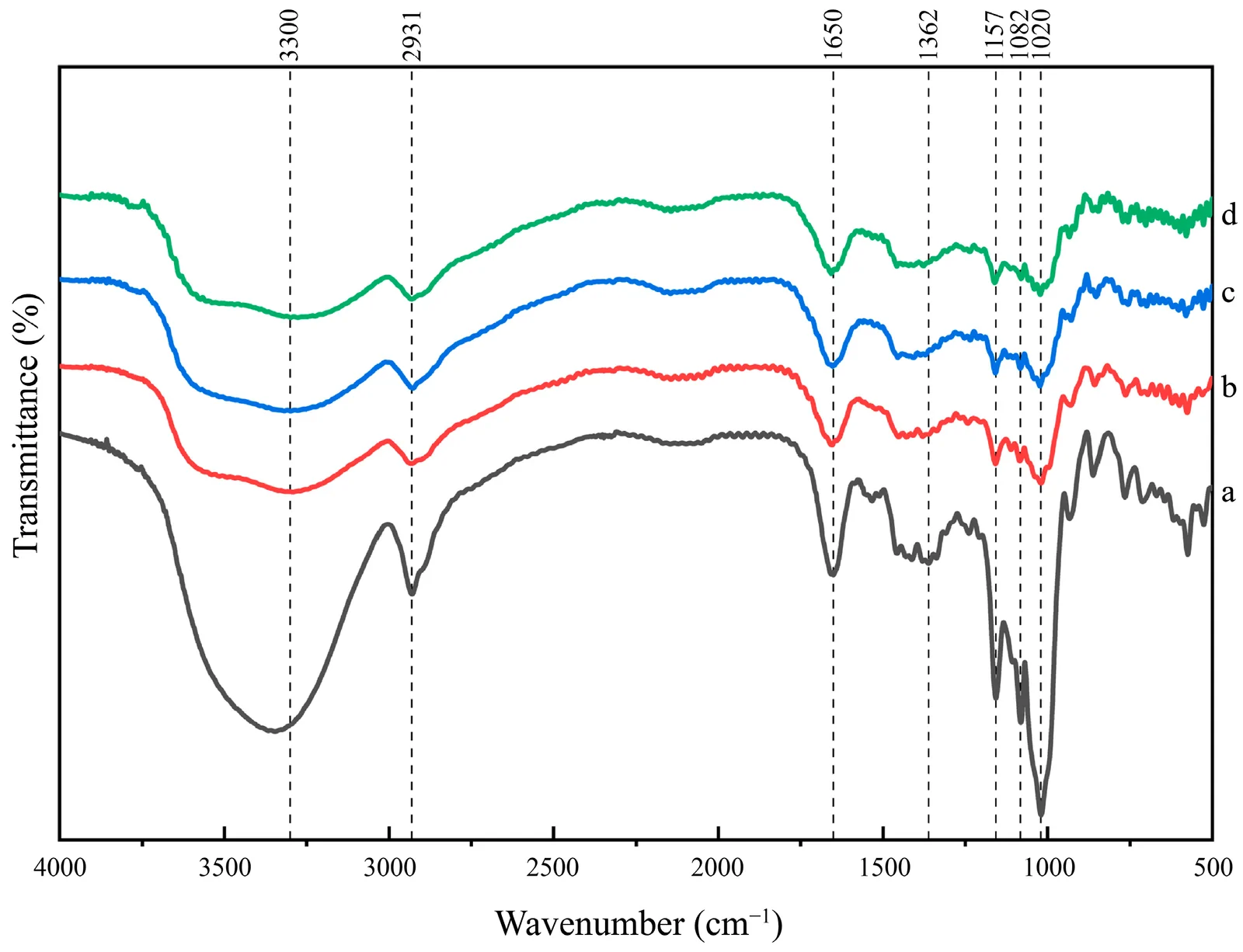
FT-IR of NR and reconstituted rice. Curves a, b, c, and d correspond to NR, MLR, NMHR, and MHR, respectively. FT-IR, Fourier transform infrared; NR, natural rice; MLR, mulberry leaf extract–low-resistant starch rice; NMHR, non-mulberry leaf extract–high-resistant starch rice; MHR, mulberry leaf extract–high-resistant starch rice.

FT-IR spectroscopy was used to characterize intermolecular interactions and changes in functional groups. As shown in Figure 3, all samples exhibited typical characteristic bands at 3300, 2931, 1650, 1362, 1157, 1082, and 1020 cm^−1^. Compared with NR, the reconstituted rice samples showed neither newly emerged characteristic absorption bands nor the disappearance of original characteristic bands; only differences in transmittance and band shape were evident. These results demonstrate that the addition of exogenous ingredients and the extrusion process do not disrupt the primary functional group structure of rice starch, which is supported by the findings of Xue et al. [38]. Moreover, no characteristic carbonyl band of Maillard reaction products (~1720 cm^−1^) was observed. Thus, no clear evidence of major new absorption bands associated with significant covalent structural modifications or Maillard reaction products was observed under the analytical conditions used in this study.

The broad absorption band at 3000–3700 cm^−1^ corresponds to O–H stretching vibration. NR showed the lowest transmittance at 3300 cm^−1^, whereas the reconstituted rice samples exhibited higher transmittance than NR, displaying a red shift in the band. Chen et al. [39] observed that hydrogen bonding between starch and phenolic hydroxyl groups of phenolic compounds exhibited a clear electronic effect, in which the electron cloud distribution of the O–H covalent bond was biased toward the oxygen atom. This reduces the electron cloud density around the hydrogen atom, which manifests as a red shift in the O–H stretching vibration absorption band in infrared spectroscopy. The band at 2931 cm^−1^ (C–H asymmetric stretching) showed no shift and only a slight increase in transmittance for the reconstituted rice, indicating that the carbon skeleton structure of the starch molecules remained intact. The 1650 cm^−1^ band (O–H bending of bound water) was more intense for NR than for the reconstituted rice groups. In the fingerprint region (900–1200 cm^−1^), all samples displayed typical bands at 1157, 1082, and 1020 cm^−1^, without shifts. Compared with reconstituted rice, NR exhibited stronger absorption intensity, indicating the occurrence of conformational changes in the starch of reconstituted rice.

### 3.4. Cooking Properties

**Figure 4:**
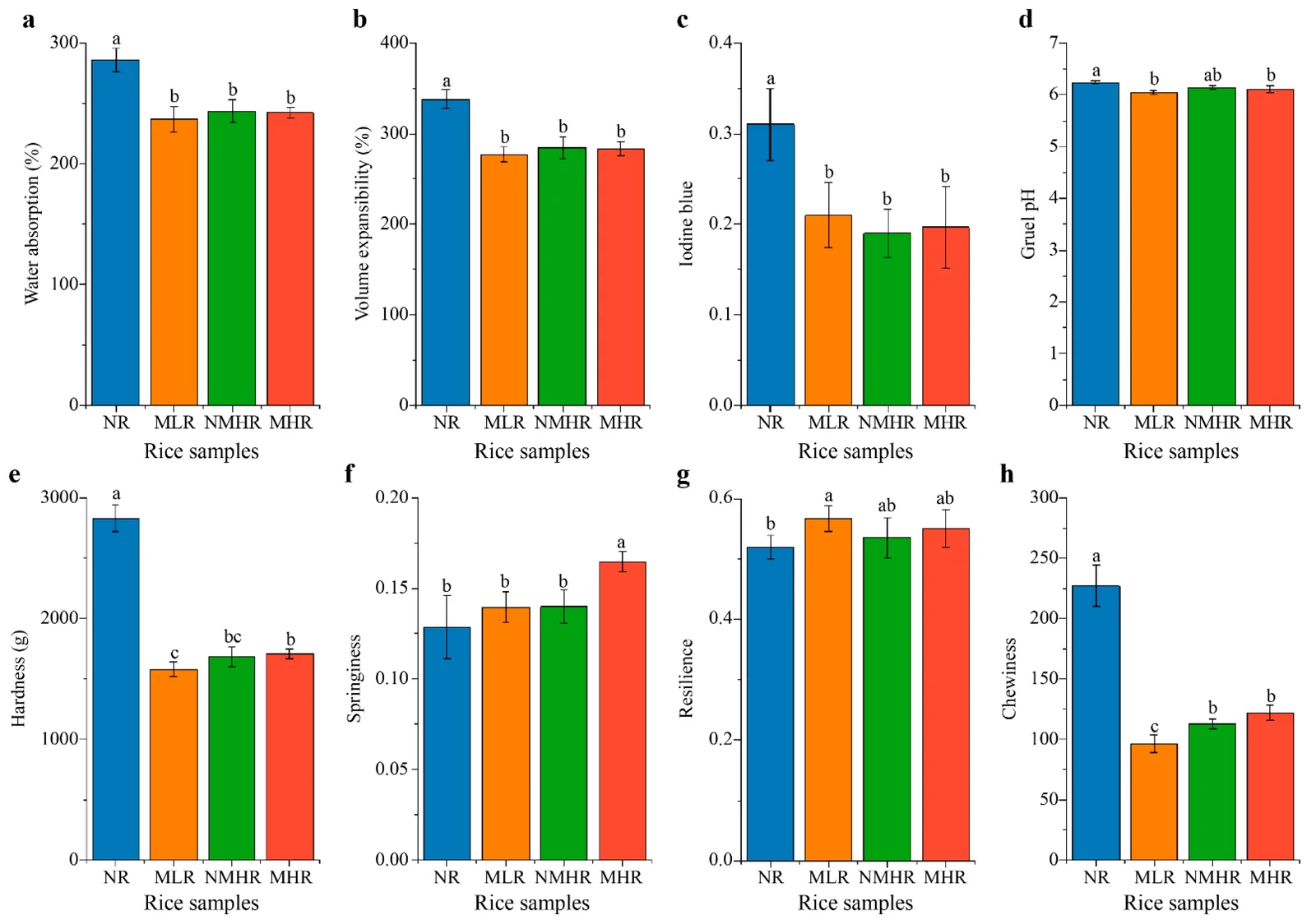
(**a**) Water absorption, (**b**) volume expansibility, (**c**) iodine blue, (**d**) gruel pH, (**e**) hardness, (**f**) springiness, (**g**) resilience, and (**h**) chewiness of NR and reconstituted rice. NR, natural rice; MLR, mulberry leaf extract–low-resistant starch rice; NMHR, non-mulberry leaf extract–high-resistant starch rice; MHR, mulberry leaf extract–high-resistant starch rice. Different letters indicate significant differences (*p* < 0.05).

Cooking properties reflect water migration and starch swelling during cooking. NR showed substantially higher WA (286.16%) and VE (338.27%) than those of the three reconstituted rice groups, the values of which remained stable at 237–244% and 277–285%, respectively (Figure 4a,b). This is attributable to the extrusion-disrupted starch crystalline structure reducing WA [25] and RS granules dispersed within the KGM gel network, which increase crosslinking density and hinder water penetration [40]. The iodine blue value of NR (0.31) was substantially higher than those of the reconstituted groups, which showed no pronounced differences among themselves (Figure 4c). This is likely because the composite gel network encapsulates starch granules, inhibiting amylose leaching during cooking. Regarding rice gruel pH (Figure 4d), the pH of NMHR (6.15) did not differ substantially from that of NR, whereas that of MLR (6.04) and MHR (6.10), both containing MLE, was markedly lower than that of NR. All pH values remained within the normal range.

### 3.5. TPA

The hardness of NR was markedly higher than that of the three reconstituted rice groups (range 1578.86–1707.96 g; Figure 4e). In particular, MLR showed the lowest value, whereas NMHR and MHR increased slightly with the RS content, without notable intergroup differences. KGM forms complexes with starch that cannot fully occupy the network structure, thereby reducing system hardness [41]. Alternatively, partial gelatinization of starch induced by extrusion, along with mechanical shear, disrupts the starch gel network structure. This softens the reconstituted rice grains and reduces their chewiness [3]. The springiness and resilience of NR were considerably lower than those of the reconstituted rice groups (Figure 4f,g). In particular, MHR exhibited the highest springiness, and MLR showed the highest resilience. These observations are attributable to the rigid support provided by RS and the network structure formed by KGM, pueraria polysaccharides, and starch. Starch fills the gaps in the KGM network, thereby forming a layered structure that enhances mechanical strength and springiness [42]. Furthermore, the interpenetrating network formed by hydrogen bonding of thermally irreversible colloids effectively improves the water-holding capacity, which enhances springiness [43]. The interaction between KGM and proteins may also lead to increased gel strength [44]. Alternatively, the amylose fraction of RS may form complexes with proteins or lipids from rice [45,46], thereby inhibiting the free movement of starch molecules. Thereby, the chewiness of the reconstituted rice groups increased slightly with increasing RS content (Figure 4h); however, it was substantially lower than that of NR (226.93). Thus, the texture of the reconstituted rice was soft and palatable.

### 3.6. Amino Acid Analysis

**Figure 5:**
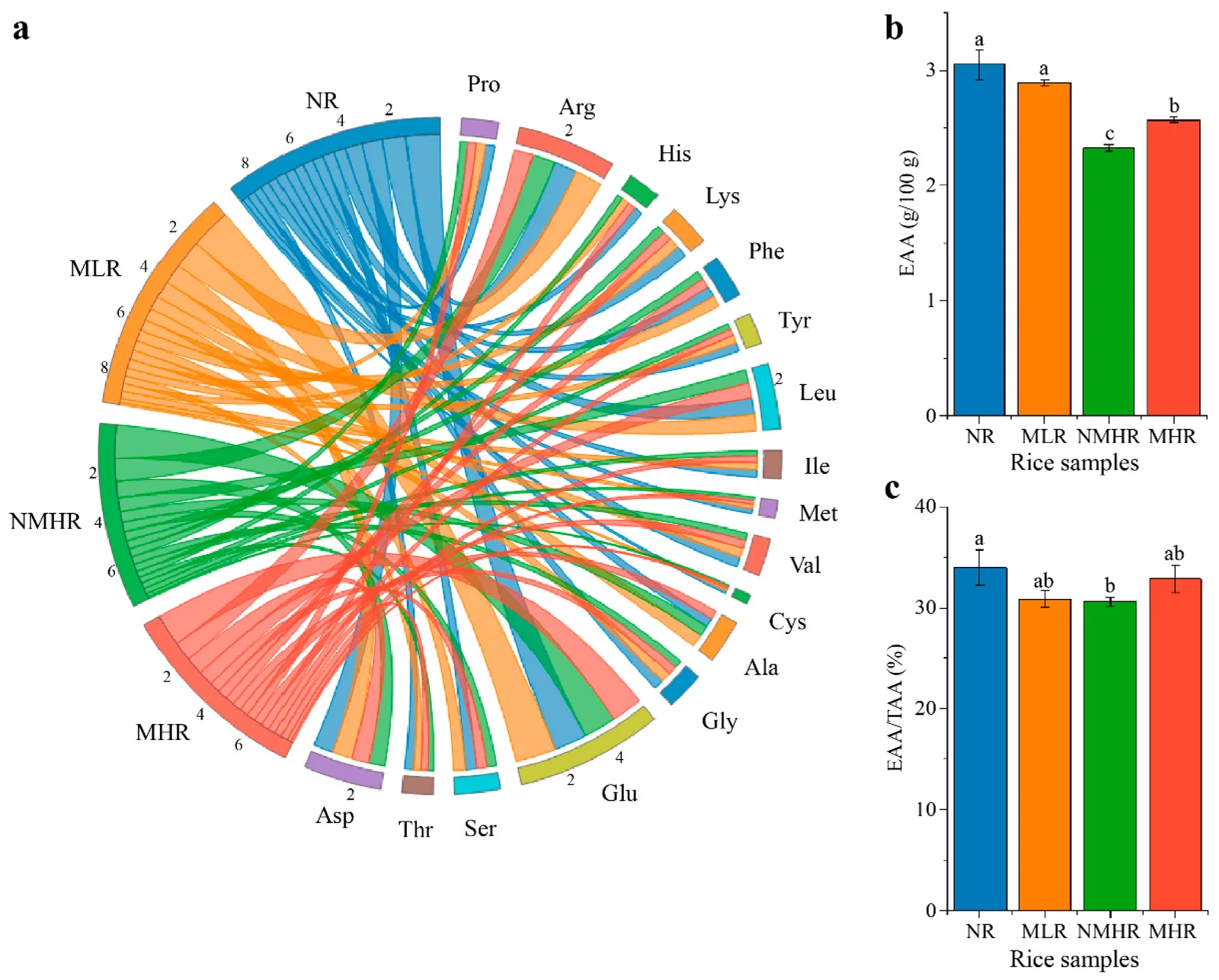
(**a**) Amino acid composition of natural and reconstituted rice. Numbers on outer arcs denote scale of amino acid content (g/100 g). (**b**) EAA content. (**c**) Proportion of EAA to TAA (EAA/TAA). EAA, essential amino acid; TAA, total amino acid; NR, natural rice; MLR, mulberry leaf extract–low-resistant starch rice; NMHR, non-mulberry leaf extract–high-resistant starch rice; MHR, mulberry leaf extract–high-resistant starch rice. Different lower-case letters above bars indicate significant differences (*p* < 0.05).

Amino acid composition is an important indicator for evaluating the nutritional value of cereal proteins. Figure 5a shows that the types of amino acids in the reconstituted rice samples were not deficient compared with those in NR. All samples contained 17 amino acids, including seven essential amino acids (EAA) (Thr, Val, Met, Ile, Leu, Phe, and Lys) and 10 non-essential amino acids (Asp, Ser, Glu, Gly, Ala, Cys, Tyr, His, Arg, and Pro), consistent with the findings of Lu et al. [47]. This indicates that the processing did not compromise the integrity of the amino acid profile. Glutamic acid was the most abundant, followed by arginine, leucine, and aspartic acid. Notably, lysine, which is the first limiting amino acid, was stably detected in all samples without an obvious degradative loss.

NR exhibited the highest absolute EAA content, whereas NMHR showed the lowest value (Figure 5b). This may be attributable to the low protein content in the functional ingredients, resulting in a dilution effect upon their addition. The EAA/total amino acid (TAA) ratios (Figure 5c) for the four sample groups ranged from 30% to 35%, which is consistent with the characteristics of high-quality rice protein.

### 3.7. Volatile Compounds

**Figure 6:**
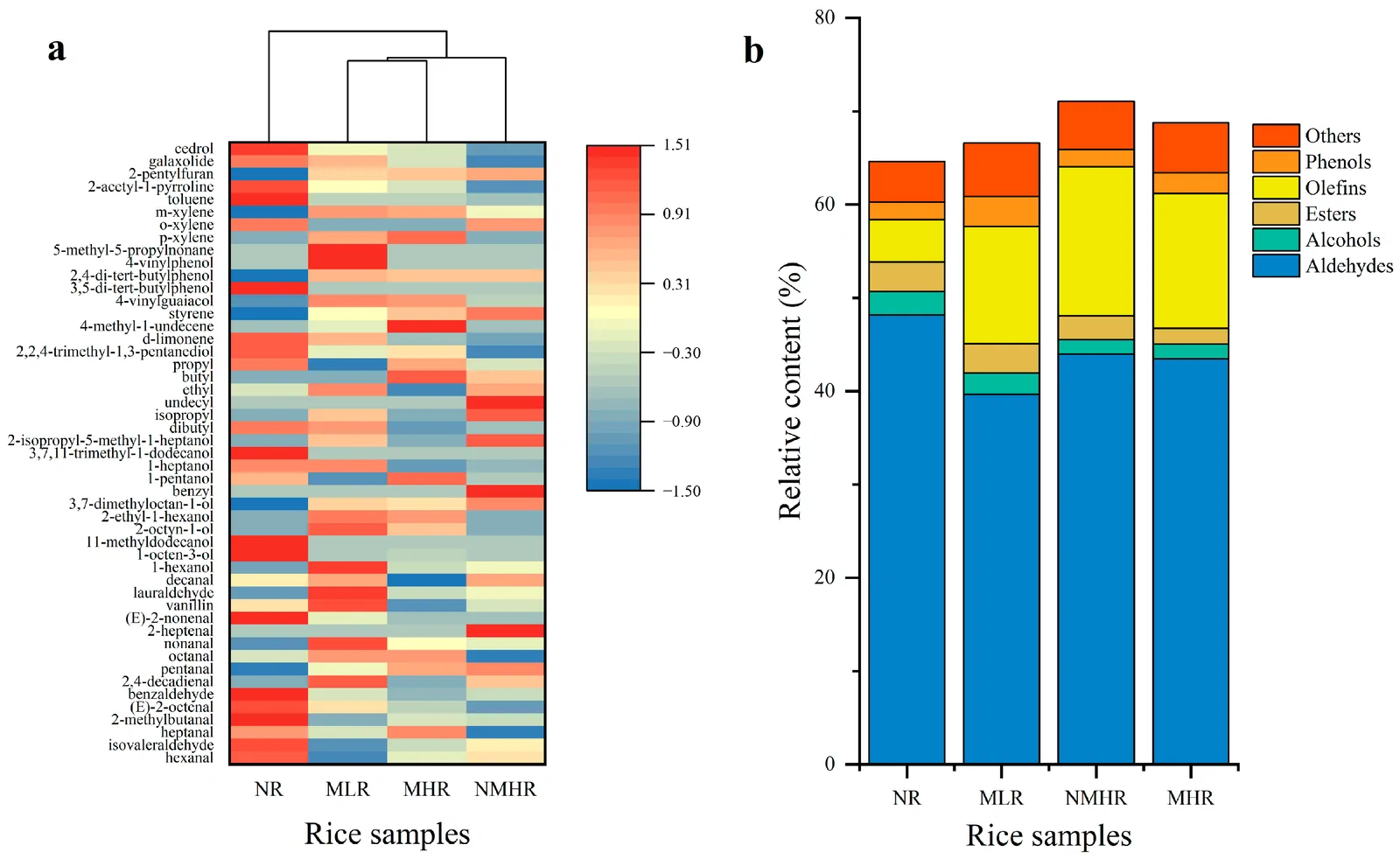
(**a**) Heatmap and (**b**) relative volatile flavor compound content in NR and reconstituted rice. NR, natural rice; MLR, mulberry leaf extract–low-resistant starch rice; NMHR, non-mulberry leaf extract–high-resistant starch rice; MHR, mulberry leaf extract–high-resistant starch rice. Different letters indicate significant differences (*p* < 0.05).

The volatile flavor compounds in cooked rice originate from diverse secondary metabolites, including aldehydes, ketones, heterocyclic compounds, and sulfur-containing derivatives. These compounds collectively render the complex aroma characteristics of cooked rice through sensory synergistic effects. The starch–protein–lipid system in rice exhibits pronounced interactions with flavor compounds that directly affect the aroma release and sensory perception of rice [48]. The cluster heatmap (Figure 6a) shows that MLR and MHR first clustered into one branch, then clustered with NMHR, and ultimately grouped with NR into a major category. This indicates that overall flavor profiles of the three reconstituted rice groups were similar to each other but substantially different from that of NR. Moreover, the addition or absence of MLE influenced the distribution of volatile compounds in the reconstituted rice. The characteristic compound 2-acetyl-1-pyrroline (2-AP), which is a signature aroma compound of fragrant rice, imparts a popcorn-like aroma to cooked rice—a key contributor to the core characteristic aroma of rice [49]. This compound was stably detected in all samples, indicating that the designed reconstituted formulations did not result in the loss of the characteristic aroma of rice.

A total of 48 volatile compounds, including aldehydes, alkenes, alcohols, esters, and phenols, were detected in cooked rice prepared from the four rice groups (Figure 6b). Aldehydes are the primary contributors to the aroma of cooked rice, mainly derived from the oxidation of unsaturated fatty acids in rice [49]. NR exhibited the highest relative aldehyde content (48.16%), whereas the reconstituted rice groups showed reduced relative aldehyde content of 39.65%, 43.97%, and 43.47%, respectively. This is attributable to the low crude fat content of the reconstituted rice, which provides fewer fatty acid substrates for oxidative degradation into aldehydes. Additionally, MLE is rich in antioxidant active substances, such as flavonoids and phenolic acids [50], which may inhibit the oxidation of unsaturated fatty acids. In addition, the relative content of alkenes and phenolic compounds was higher in reconstituted rice groups than in NR. Notably, MLR and MHR had higher content of phenolic compounds than NMHR without MLE. The increased relative content of alkenes and phenols in the reconstituted rice mainly originated from the terpenes and phenols abundantly present in the functional ingredients.

### 3.8. Sensory Evaluation

**Figure 7:**
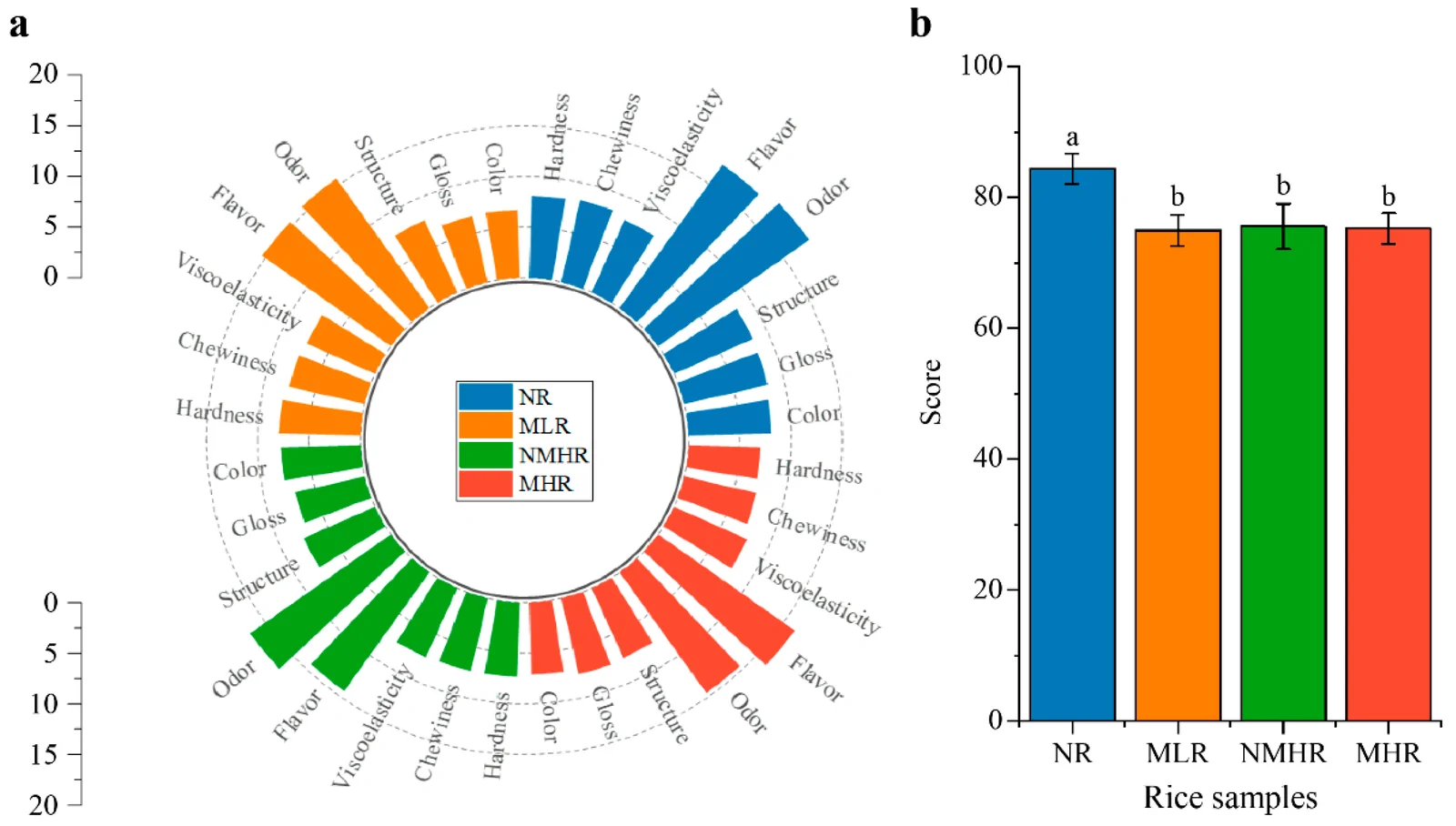
(**a**) Individual item scores and (**b**) total scores for sensory evaluation of cooked rice prepared from NR and reconstituted rice. NR, natural rice; MLR, mulberry leaf extract–low-resistant starch rice; NMHR, non-mulberry leaf extract–high-resistant starch rice; MHR, mulberry leaf extract–high-resistant starch rice. Different letters indicate significant differences (*p* < 0.05).

The sensory scores of cooked rice prepared from NR and reconstituted rice are shown in Figure 7. NR had the highest total sensory score (84.4), which aligns with the cooking quality, flavor, and texture of natural rice, as well as with the long-established dietary habits of panelists. The three reconstituted rice groups showed no pronounced differences in total sensory scores, all of which exceeded 70. Although no unacceptable sensory defects were reported, indicating that the formulation and extrusion processing achieved functional enhancement of reconstituted rice while maintaining palatability, the reconstituted rice exhibited some differences in appearance compared with NR. In terms of appearance, NR received the highest scores for color, luster, and structural integrity. The reconstituted rice exhibited darker coloration due to pigments from MLE, and the bubbles formed during extrusion affected the appearance and structure, leading to low scores. In terms of odor and flavor, NR possessed a pure and natural rice aroma and received the highest score. The odor and flavor scores of NMHR cooked rice were slightly higher than those of MLR and MHR. This is because the mulberry leaf components imparted an additional odor to the rice that does not align with panelists’ long-established sensory memory of rice. In terms of texture, the reconstituted rice groups received lower scores than those of NR for hardness and chewiness. Although the total sensory scores of the reconstituted rice were lower than those of NR, no pronounced sensory defects were noted for any of the samples. Hence, the samples achieved good palatability alongside functional enhancement, demonstrating potential for industrial application.

### 3.9. In Vitro Digestion and eGI

**Figure 8:**
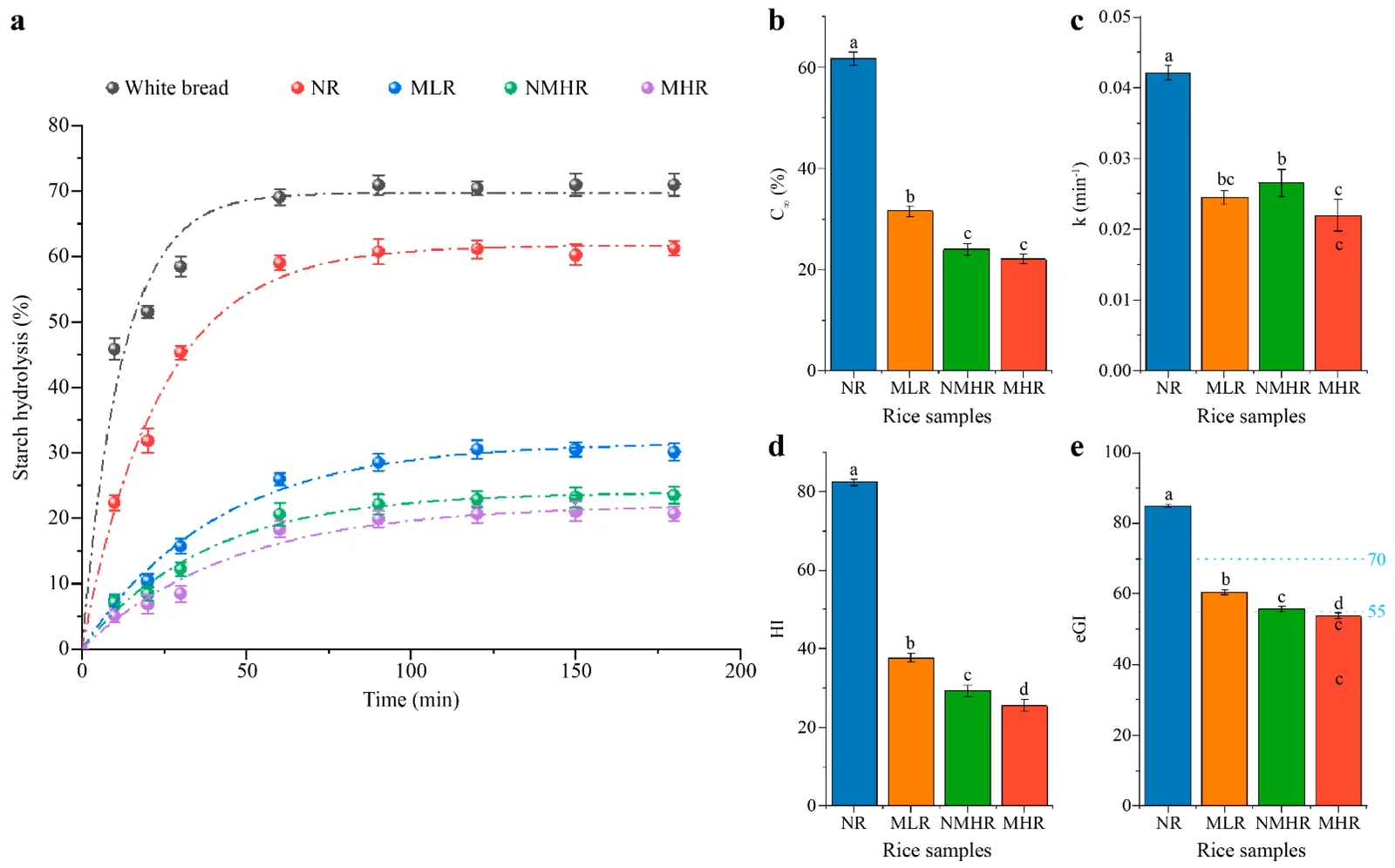
(**a**) Digestion curves, (**b**) final hydrolysis extent (C∞), (**c**) kinetic constant (k), (**d**) hydrolysis index (HI), and (**e**) estimated glycemic index (eGI) for different samples. NR, natural rice; MLR, mulberry leaf extract–low-resistant starch rice; NMHR, non-mulberry leaf extract–high-resistant starch rice; MHR, mulberry leaf extract–high-resistant starch rice. Different letters indicate significant differences (*p* < 0.05).

In vitro digestion is a method used to evaluate the blood glucose regulation potential of foods. The starch hydrolysis curves (Figure 8) showed that the hydrolysis rates of all samples exhibited first-order kinetic characteristics, with a rapid initial increase followed by a plateau. The white bread exhibited the fastest hydrolysis, with the hydrolysis rate exceeding 50% at 20 min, hydrolysis equilibrium at 90 min, and a final hydrolysis rate stabilized at approximately 70%. The hydrolysis rate for NR was lower than that for white bread, with a final hydrolysis rate of 61.65%. The hydrolysis rates and final hydrolysis rates for the three reconstituted rice groups were substantially lower than those for NR, and the magnitude of reduction closely aligned with the gradient of the functional components in the formulation, thereby exhibiting the order MLR > NMHR > MHR. The kinetic parameters indicated that NR had the largest hydrolysis rate constant k (4.21 × 10^−2^ min^−1^), which was markedly higher than those of all reconstituted rice groups, with MHR showing the lowest k value. The final hydrolysis extent (C∞) for the reconstituted rice also decreased in a gradient manner depending on functional ingredient concentration. Assuming that the GI value for the standard test food (white bread) is 100, foods can be classified as having high (GI > 70), medium (56–69), or low (GI < 55) GI based on the extent of postprandial blood glucose elevation [51]. MLR and NMHR exhibited eGI values within the medium-GI range, whereas MHR exhibited an eGI value within the low-GI range, which is suggestive of its potential to meet the low-GI food standard. These results indicate that adding functional ingredients effectively reduces the GI value of reconstituted rice. Notably, RS is a novel type of dietary fiber that is not digested or absorbed in the small intestine but can be utilized by colonic microorganisms to produce short-chain fatty acids. The addition of RS to reconstituted rice effectively slows the digestion rate of rice and stabilizes postprandial fluctuations in blood glucose levels [52]. Furthermore, 1-deoxynojirimycin from MLE exhibits strong inhibitory potential against α-amylase, glucoamylase, and sucrase activities, thereby effectively suppressing the elevation in postprandial blood glucose levels [53,54]. In addition, proteins form a protective physical barrier on starch granules, which reduces starch gelatinization, gelation, and the viscosity of the food matrix. This decreases the rate of starch hydrolysis and digestion [55]. Endogenous proteins, fats, and starch in the raw materials inevitably interact during high-temperature extrusion to form complexes [56,57]. Given that the three formulations simultaneously differ in their RS content, MLE supplementation, and minor KP adjustment, the decreased starch digestibility of the reconstituted rice should be attributed to the combined effects of multiple functional ingredients rather than to the independent effect of any single component. The individual contributions of RS, MLE, and their potential interaction cannot be fully disentangled with the present combinatorial formulation design. Although these complex interactions are not yet fully understood, they may exert a retarding effect on starch digestion. Therefore, through a synergistic formulation strategy of physical barrier–enzyme inhibition–substrate regulation, the development of reconstituted rice with low glycemic potential, as estimated by in vitro digestion tests, was successfully achieved.

## 4. Limitations

This study had certain limitations. First, the extrusion processing parameters were fixed based on preliminary trials, and the effects of key parameters (e.g., temperature, screw speed, feed moisture) on product quality were not systematically explored. Second, the combinatorial formulation design of this study could not independently quantify the respective contributions of KP, MLE, PP, RS, and their interactions to the reduction in starch digestibility. Third, the GI-lowering effect was only evaluated using an in vitro simulated gastrointestinal digestion model, which cannot fully replicate the complex physiological environment of the human gastrointestinal tract, including dynamic digestive enzyme secretion, gut microbiota-mediated metabolism, and interindividual metabolic heterogeneity. Finally, from a quality perspective, we did not conduct a systematic assessment of the long-term stability of functionally reconstituted rice during storage. Future studies should systematically optimize extrusion parameters via response surface methodology to further improve the comprehensive quality and low-GI function of reconstituted rice; adopt single-factor control or factorial designs to clarify the independent contribution of each functional ingredient; conduct animal experiments and human clinical trials to validate its in vivo glycemic regulatory effects and long-term health benefits; and investigate the quality change rules during storage to provide a more solid scientific basis for industrial production and market distribution of functional reconstituted rice.

## 5. Conclusions

In this study, we investigated the effects of KP, MLE, PP, and RS on the microstructure, physicochemical properties, nutritional quality, flavor, and in vitro starch digestibility of reconstituted rice and elucidated the synergistic mechanism underlying the low eGI effect. The functional ingredients reshaped the nutritional composition by reducing available carbohydrates, proteins, and fats while increasing dietary fiber and RS. This essentially provided the material basis for low-eGI rice. The colloidal network formed by KGM, starch, and protein restricted WA and VE during cooking and improved the texture. The product retained the complete amino acid profile and characteristic flavor of raw rice while incorporating unique aroma compounds from the functional ingredients. This enriches flavor complexity, achieving synergistic enhancement of nutritional and eating qualities. In vitro digestion confirmed that the combined ingredients greatly disrupted starch hydrolysis and lowered the final hydrolysis extent through a triple pathway consisting of a physical barrier, enzyme activity inhibition, and substrate regulation. The three developed formulations, with different eGI gradients, possessed both nutritional quality and palatability. Hence, these rice formulations not only have the potential to serve as daily staple foods for people with diabetes, obesity, or glucose impairment, but can also be used for blood glucose management across the lifespan of healthy populations, demonstrating prospects for broad industrial application. Future studies should include in vivo animal experiments and human clinical trials to verify the long-term glycemic regulatory effects and health benefits. This will provide comprehensive scientific evidence for market promotion.

## Figures and Tables

**Table 1:** Formulation of reconstituted rice.

Formulation	NR (%)	KP (%)	MLE (%)	PP (%)	RS (%)
MLE–low-RS rice (MLR)	86.5	9.0	0.5	2.0	2.0
Non-MLE–high-RS rice (NMHR)	82.1	8.5	-	2.0	7.4
MLE–high-RS rice (MHR)	80.0	9.0	0.5	2.0	8.5

NR, natural rice; KP, konjac powder; MLE, mulberry leaf extract; PP, pueraria powder; RS, resistant starch.

**Table 2:** Sensory evaluation criteria for cooked rice.

Attribute (Score)	Sub-Indicator (Score)	Criteria	Score Range
Appearance (30)	Color (10)	Uniform	8–10
		Relatively uniform	4–7
		Uneven	0–3
	Gloss (10)	Attractive gloss	8–10
		Slight gloss	4–7
		Without gloss	0–3
	Structure (10)	Compact and intact	8–10
		Relatively compact and intact	4–7
		Severely cracked	0–3
Aroma (20)	Odor (20)	No off-odor	15–20
		No obvious off-odor	8–14
		Unpleasant smell	0–7
Taste (20)	Flavor (20)	Pronounced rice flavor	15–20
		Mild rice flavor	8–14
		No rice flavor	0–7
Texture (30)	Viscoelasticity (10)	Sticky, not adhesive to teeth	8–10
		Slightly sticky, not easily adhesive to teeth	4–7
		Non-sticky, adhesive to teeth	0–3
	Chewiness (10)	Good chewiness	8–10
		Little chewiness	4–7
		No chewiness	0–3
	Hardness (10)	Moderate	8–10
		Slightly hard or soft	4–7
		Very hard or very soft	0–3

## Data Availability

The original contributions presented in this study are included in the article. Further inquiries can be directed to the corresponding authors.

## Abbreviations

The following abbreviations are used in this manuscript:

GI	Glycemic index
KP	Konjac powder
MLE	Mulberry leaf extract
PP	Pueraria powder
RS	Resistant starch
NR	Natural rice
MLR	MLE-low RS rice
NMHR	Non-MLE-high RS rice
MHR	MLE-high RS rice
WA	Water absorption
VE	Volume expansibility
TPA	Texture profile analysis
GC-MS	Gas chromatography–mass spectrometry
eGI	Estimated glycemic index
FT-IR	Fourier transform infrared
EAA	Essential amino acid
TAA	Total amino acid

## References

[1] Yang W., Zheng Y., Sun W., Chen S., Liu D., Zhang H., Fang H., Tian J., Ye X. (2020). Effect of extrusion processing on the microstructure and in vitro digestibility of broken rice. LWT.

[2] Goufo P., Trindade H. (2014). Rice antioxidants: Phenolic acids, flavonoids, anthocyanins, proanthocyanidins, tocopherols, tocotrienols, γ-oryzanol, and phytic acid. Food Sci. Nutr..

[3] Gao M., Ma C., Xu Y., Liu Y., Wang B., Zhang G., Xu X., Yang Y., Zhang N. (2026). Anti-digestive reconstituted rice prepared by extrusion of modified japonica rice starch: Structural, physicochemical, and digestive properties. Int. J. Biol. Macromol..

[4] Dalbhagat C.G., Mahato D.K., Mishra H.N. (2019). Effect of extrusion processing on physicochemical, functional and nutritional characteristics of rice and rice-based products: A review. Trends Food Sci. Technol..

[5] Ganachari A., Nidoni U., Hiregoudar S., Ramappa K.T., Naik N., Vanishree S., Mathad P.F. (2022). Development of rice analogues fortified with iron, folic acid and vitamin A. J. Food Sci. Technol..

[6] Kuong K., Tor P., Perignon M., Fiorentino M., Chamnan C., Berger J., Burja K., Dijkhuizen M.A., Parker M., Roos N. (2019). Multi-micronutrient fortified rice improved serum zinc and folate concentrations of Cambodian school children. A double-blinded cluster-randomized controlled trial. Nutrients.

[7] Wang R.J., Tang J.E., Chen Y., Gao J.G. (2015). Dietary fiber, whole grains, carbohydrate, glycemic index, and glycemic load in relation to risk of prostate cancer. OncoTargets Ther..

[8] Yan C., Kim S.-R., Ruiz D.R., Farmer J.R. (2022). Microencapsulation for food applications: A review. ACS Appl. Bio Mater..

[9] Köhler J., Teupser D., Elsässer A., Weingärtner O. (2017). Plant sterol enriched functional food and atherosclerosis. Br. J. Pharmacol..

[10] Zhang Q., Zhang L., Li X., He Z., Chen S., Zhang D. (2021). Response surface methodology for optimizing twin-screw prepared *Cistanche deserticola*—Potato composite rice. Starch Stärke.

[11] Sun Y., Xu X., Zhang Q., Zhang D., Xie X., Zhou H., Wu Z., Liu R., Pang J. (2023). Review of konjac glucomannan structure, properties, gelation mechanism, and application in medical biology. Polymers.

[12] Laignier F., Akutsu R.C.C.A., Maldonade I.R., Bertoldo Pacheco M.T., Silva V.S.N., Mendonça M.A., Zandonadi R.P., Raposo A., Botelho R.B.A. (2021). Amorphophallus konjac: A novel alternative flour on gluten-free bread. Foods.

[13] Das M., Santra S., Chakraborty M., Rajan N., Sarvanabhupathy S., Anusha B., Biswas P., Banerjee R. (2024). Resistant starch: Insights into better health and metabolism. Biocatal. Agric. Biotechnol..

[14] Li X., Chen R., Wen J., Ji R., Chen X., Cao Y., Yu Y., Zhao C. (2024). The mechanisms in the gut microbiota regulation and type 2 diabetes therapeutic activity of resistant starches. Int. J. Biol. Macromol..

[15] Li H., Zhang L., Li J., Wu Q., Qian L., He J., Ni Y., Kovatcheva-Datchary P., Yuan R., Liu S. (2024). Resistant starch intake facilitates weight loss in humans by reshaping the gut microbiota. Nat. Metab..

[16] Rao Y., Wen Q., Liu R., He M., Jiang Z., Qian K., Zhou C., Li J., Du H., Ouyang H. (2020). PL-S2, a homogeneous polysaccharide from radix Puerariae lobatae, attenuates hyperlipidemia via farnesoid X receptor (FXR) pathway-modulated bile acid metabolism. Int. J. Biol. Macromol..

[17] Suriyaprom S., Srisai P., Intachaisri V., Kaewkod T., Pekkoh J., Desvaux M., Tragoolpua Y. (2023). Antioxidant and anti-inflammatory activity on LPS-stimulated RAW 264.7 macrophage cells of white mulberry (*Morus alba* L.) leaf extracts. Molecules.

[18] Thabti I., Elfalleh W., Tlili N., Ziadi M., Campos M.G., Ferchichi A. (2014). Phenols, flavonoids, and antioxidant and antibacterial activity of leaves and stem bark of *Morus* species. Int. J. Food Prop..

[19] Li Q., Wang Y., Dai Y., Shen W., Liao S., Zou Y. (2019). 1-deoxynojirimycin modulates glucose homeostasis by regulating the combination of IR-GlUT4 and ADIPO-GLUT4 pathways in 3T3-L1 adipocytes. Mol. Biol. Rep..

[20] Liu S., Lu H., Chen W., Ma X., Liu J., Xia K., Zhou Z., Han X., Wu Y., Liu J. (2022). Preparation and evaluation of low glycemic index reconstituted rice. Food Sci. Technol. Res..

[21] (2016). National Food Safety Standard—Determination of Moisture in Foods.

[22] (2016). National Food Safety Standard—Determination of Protein in Foods.

[23] (2016). National Food Safety Standard—Determination of Fat in Foods.

[24] (2014). National Food Safety Standard—Determination of Dietary Fiber in Foods.

[25] Liao Z., Li T., Chen H., Li S., Wu J., Wang F., Li X. (2025). Effects of exogenous amyloid protein fibril aggregates on in vitro digestibility, structural properties and retrogradation of starch in extruded reconstituted rice. Carbohydr. Polym..

[26] Yang X., Ma L., Yu P., Qiao Y., Feng Z., Bai J., Zhou R., Wang C., Cai J. (2025). The comparative evaluation of the quality of brown rice by plasma treatment and milling treatment: Appearance, cooking characteristics, texture characteristics, and nutrient composition. J. Cereal Sci..

[27] Tang Y., Liu X., Jiang F., Shen W., Chen X., Jin W. (2026). Milling dynamics of long-grain rice and characterization of its bran retention using x-ray micro-CT reconstruction. J. Cereal Sci..

[28] (2016). National Food Safety Standard—Determination of Amino Acids in Foods.

[29] Kang L., Luo J., Su Z., Zhou L., Xie Q., Li G. (2024). Effect of Sprouted Buckwheat on Glycemic Index and Quality of Reconstituted Rice. Foods.

[30] (2008). Inspection of Grain and Oils—Method for Sensory Evaluation of Paddy or Rice Cooking and Eating Quality.

[31] Minekus M., Alminger M., Alvito P., Ballance S., Bohn T., Bourlieu C., Carrière F., Boutrou R., Corredig M., Dupont D. (2014). A standardised static in vitro digestion method suitable for food—An international consensus. Food Funct..

[32] Butterworth P.J., Warren F.J., Grassby T., Patel H., Ellis P.R. (2012). Analysis of starch amylolysis using plots for first-order kinetics. Carbohydr. Polym..

[33] Goñi I., Garcia-Alonso A., Saura-Calixto F. (1997). A starch hydrolysis procedure to estimate glycemic index. Nutr. Res..

[34] Mohamed I.O. (2023). Interaction of starch with some food macromolecules during the extrusion process and its effect on modulating physicochemical and digestible properties. A review. Carbohydr. Polym. Technol. Appl..

[35] Wu C., Wu F., Zhang Y., Liu F., Luan G. (2025). Effect of extruded soybean okara on the texture, rheology, and structural properties of high-fiber composite dough. Food Chem..

[36] Wang K., Ma J., Wang L., Yue X., Ma X., Huo J., Duan Y., Wang P., Yu X., Xiao Z. (2024). Insight into the relationship between the starch crystalline structure and textural quality and physicochemical properties of reconstituted rice: Influence of feed moisture content. Int. J. Biol. Macromol..

[37] Gutiérrez Á.L., Villanueva M., Rico D., Harasym J., Ronda F., Martín-Diana A.B., Caballero P.A. (2023). Valorisation of buckwheat by-product as a health-promoting ingredient rich in fibre for the formulation of gluten-free bread. Foods.

[38] Xue S., Cui Z., Yang L., Wang S., He Y., Zhang Y., Liu H. (2024). The effect of okara on physical–chemical characteristics and starch hydrolysis of extruded reconstituted rice in vitro. J. Funct. Foods.

[39] Chen N., Feng Z.-J., Gao H.-X., He Q., Zeng W.-C. (2022). Effects of phenols with different structure characteristics on properties of potato starch: Action rule and molecular mechanism. J. Food Process. Preserv..

[40] Su X., Xu Y., Wang Z., Zhou H., Xu B. (2024). Physicochemical, interactions, and morphology of l-arginine/konjac glucomannan gel: Effects of modified starches. J. Food Eng..

[41] Meng K., Gao H., Zeng J., Zhao J., Qin Y., Li G., Su T. (2021). Rheological and microstructural characterization of wheat dough formulated with konjac glucomannan. J. Sci. Food Agric..

[42] Liang Y., Yu H., Song K., Zhou X., Liu L., Guo J. (2025). Optimal preparation process and in vitro digestion characteristics of konjac glucomannan and kudzu root resistant starch composite gel. Food Biosci..

[43] Wang J., Liu Y., Zhao M., Sun Q., Li M., Wang Y., Zhang Y., Xie F. (2024). Effect of curdlan addition and thermal sterilization on the structural and properties of rice starch gel. Int. J. Biol. Macromol..

[44] Jian W., Wu H., Wu L., Wu Y., Jia L., Pang J., Sun Y.M. (2016). Effect of molecular characteristics of konjac glucomannan on gelling and rheological properties of *Tilapia* myofibrillar protein. Carbohydr. Polym..

[45] Wang L., Zhang L., Wang H., Ai L., Xiong W. (2020). Insight into protein-starch ratio on the gelatinization and retrogradation characteristics of reconstituted rice flour. Int. J. Biol. Macromol..

[46] Zhao X., Li X., Guo R., Wang X., Zeng L., Wen X., Huang Q. (2023). Different oil-modified cross-linked starches: In vitro digestibility and its relationship with their structural and rheological characteristics. Food Chem..

[47] Lu X., Chang R., Lu H., Ma R., Qiu L., Tian Y. (2021). Effect of amino acids composing rice protein on rice starch digestibility. LWT.

[48] Lina G., Min Z. (2022). Formation and release of cooked rice aroma. J. Cereal Sci..

[49] Hu X., Lu L., Guo Z., Zhu Z. (2020). Volatile compounds, affecting factors and evaluation methods for rice aroma: A review. Trends Food Sci. Technol..

[50] Zhao C., Li T., Zhang C., Li H., Wang Y., Li C., Wang Z., Zhao M., Shen M., Zhao W. (2025). Drying methods affect nutritional value, amino acids, bioactive compounds, and in vitro function of extract in mulberry leaves. Food Chem..

[51] Jeevarathinam G., Ramniwas S., Singh P., Rustagi S., Mohammed Basheeruddin Asdaq S., Pandiselvam R. (2024). Macromolecular, thermal, and nonthermal technologies for reduction of glycemic index in food-A review. Food Chem..

[52] Wang J., Wang C., Yu J., Yang Y., Copeland L., Wang S. (2025). A novel composite resistant starch with improved prebiotic functions. Food Hydrocoll..

[53] Qiao Y., Ito M., Kimura T., Ikeuchi T., Takita T., Yasukawa K. (2021). Inhibitory effect of *Morus australis* leaf extract and its component iminosugars on intestinal carbohydrate-digesting enzymes. J. Biosci. Bioeng..

[54] Zhang X., Yuan P., Zhou F., Xia K., Liu J., Yang Z., Zhou F., Duan S., Ma C., Liu J. (2025). Establishment of innovative preparation technology for multi-targeted extracts of mulberry leaves and insight into inhibition mechanism of α-glucosidase: In vitro digestion, kinetics, and spectroscopic analysis. J. Future Foods.

[55] Garcia-Valle D.E., Bello-Pérez L.A., Agama-Acevedo E., Alvarez-Ramirez J. (2021). Structural characteristics and in vitro starch digestibility of pasta made with durum wheat Semolina and chickpea flour. LWT.

[56] Chen Q., Zhang J., Liu H., Li T., Wang Q. (2023). Mechanism of high-moisture extruded protein fibrous structure formation based on the interactions among pea protein, amylopectin, and stearic acid. Food Hydrocoll..

[57] Muñoz-Pabon K.S., Parra-Polanco A.S., Roa-Acosta D.F., Hoyos-Concha J.L., Bravo-Gomez J.E. (2022). Physical and paste properties comparison of four snacks produced by high protein quinoa flour extrusion cooking. Front. Sustain. Food Syst..

